# Effect of Low Temperature on Changes in AGP Distribution during Development of *Bellis perennis* Ovules and Anthers

**DOI:** 10.3390/cells10081880

**Published:** 2021-07-24

**Authors:** Agata Leszczuk, Ewa Szczuka, Kinga Lewtak, Barbara Chudzik, Artur Zdunek

**Affiliations:** 1 Institute of Agrophysics, Polish Academy of Sciences, 20-290 Lublin, Poland; a.zdunek@ipan.lublin.pl; 2 Department of Cell Biology, Institute of Biological Sciences, Maria Curie-Skłodowska University, 20-033 Lublin, Poland; ewa.szczuka@poczta.umcs.lublin.pl (E.S.); kinga.lewtak@poczta.umcs.lublin.pl (K.L.); 3 Department of Biological and Environmental Education with Zoological Museum, Institute of Biological Sciences, Maria Curie-Skłodowska University, 20-033 Lublin, Poland; bchudzik@poczta.umcs.lublin.pl

**Keywords:** anther, arabinogalactan proteins, cell wall, gametophyte, immunocytochemistry, plant development, plant reproduction, ovule

## Abstract

Arabinogalactan proteins (AGPs) are a class of heavily glycosylated proteins occurring as a structural element of the cell wall-plasma membrane continuum. The features of AGPs described earlier suggest that the proteins may be implicated in plant adaptation to stress conditions in important developmental phases during the plant reproduction process. In this paper, the microscopic and immunocytochemical studies conducted using specific antibodies (JIM13, JIM15, MAC207) recognizing the carbohydrate chains of AGPs showed significant changes in the AGP distribution in female and male reproductive structures during the first stages of *Bellis perennis* development. In typical conditions, AGPs are characterized by a specific persistent spatio-temporal pattern of distribution. AGP epitopes are visible in the cell walls of somatic cells and in the megasporocyte walls, megaspores, and embryo sac at every stage of formation. During development in stress conditions, the AGP localization is altered, and AGPs entirely disappear in the embryo sac wall. In the case of male development, AGPs are present in the tapetum, microsporocytes, and microspores in normal conditions. In response to development at lower temperature, AGPs are localized in the common wall of microspores and in mature pollen grains. Additionally, they are accumulated in remnants of tapetum cells.

## 1. Introduction

Arabinogalactan proteins (AGPs) are a group of structural proteins present in the extracellular matrix in plant cells [1,2,3]. AGPs are a class of the subfamily of hydroxyproline-rich glycoproteins (HRGPs) characterized by a specific molecular structure. Its protein moiety contains hydroxyproline, alanine, serine, threonine, and glycine, and constitutes around 10% of the total molecular mass. The other considerable parts of the AGP molecule are carbohydrate chains composed of arabinose and galactose-rich polysaccharide units with varying amounts of rhamnose, glucuronic acid, and fucose. The *O*-glycosylation of AGPs on Hyp residues constitutes the basis of their essential roles in plant metabolism. Properly formed carbohydrate chains of AGPs have an effect on proper action of AGPs and are thus involved in the establishment of cell wall-plasma membrane continuum and cross-linking with other cell-wall constituents [4,5]. AGPs are involved in assembly of the ARABINOXYLAN PECTIN ARABINOGALACTAN PROTEIN 1 complex—APAP1 [5], which is currently one of the most novel models of the cell wall structure and the type of binding components. In the APAP1 complex, AGPs play a significant role as constituents connecting arabinoxylan, rhamnogalacturonan I, and homogalacturonan, which are covalently linked through the rhamnosyl residue in the AGP carbohydrate domain [5]. Moreover, a unique feature of AGPs is the presence of a C-terminal glycosylphosphatidylinositol (GPI anchor) sequence that allows their anchoring to the plasma membrane [4]. GPI anchored proteins (GAPs) are regarded as candidates for plasma membrane-cell wall interactions [6].

AGPs are assumed to play roles in the plant sexual reproduction process [7,8,9]. They have been shown to be present at many stages of this process [10]. AGPs were found to appear during microsporogenesis as a precise molecular marker of the particular stages of anther development [3,11]. Similarly, our previous work [12] described the temporal and spatial pattern of the distribution of AGP epitopes during male gametophyte development in *Bellis perennis*. It was found that AGP epitopes occurred in the cell wall surrounding microsporocytes until the end of meiosis in the four newly formed microspores. Moreover, it is certain that the presence of AGPs is correlated with female gametophyte development [13,14]. Genetic analyses of the *AGP18* gene performed by Acosta-García and Vielle-Calzada [15] confirmed their participation in the beginning of megasporogenesis as molecules important for the interaction between somatic and generative cells. Furthermore, the changes observed in their localization during megasporogenesis indicate that AGPs are present also during the subsequent initiation of the development of the embryo sac [16,17]. Also, AGP epitopes are visible in the mature embryo sac stage [18] and in the transmission tissue in the pathway of pollen tube [19]. Comprehensive studies of AGPs in the developmental process of *Fragaria x ananassa* from gametophyte development [13], and embryo sac formation [20] to pollen tube growth and fertilization [21] allow concluding that AGPs are present at every stage of plant reproduction.

Additionally, it is well known that changeable temperature conditions may influence plant development, including changes in the cell wall metabolism [22]. In the case of developmental processes, low temperature stress has several major effects on reproductive tissues, i.e., asynchrony between male and female reproductive development, early or delayed flowering, defects in parental tissue, and defects in male and female structures such as reduced ovule size, reduced ovule viability, missing embryo sacs, disruption of sugar metabolism in the tapetum ultimately abolishing starch accumulation in pollen grains, and shortened pollen tubes [23,24]. The elucidation of the mechanisms employed by different plants to cope with stress during the reproduction process is critical for the subsequent stages of their development and growth.

Therefore, the aim of the present study was to examine the effect of low temperature stress on the arrangement of AGP epitopes during the developmental process in *B. perennis,* which is a species belonging to the *Asteraceae*, commonly used in embryological research. The biennial *B. perennis* is very resistant to low temperatures, as evidenced by its flowering throughout the year, except for period when there is a thick snow cover on the ground. In this paper, we describe the changes in the AGP distribution pattern resulting from low temperature treatment as a continuation of our previous papers, in which we analyzed the spatio-temporal distribution of AGPs in female and male gametophytes in typical normal conditions. It is well known that the properties and roles of AGPs are an effect of their molecular structure and the presence of a glyco-moiety with carbohydrate chains characterized by polydispersity due to the different numbers of repetitive AG subunits [4]. Thus, JIM13, JIM15, and MAC207, which recognized carbohydrate units of AGPs, were selected for immunofluorescence labeling of AGPs in *B. perennis.* We observed changes in the distribution of these epitopes in both somatic- and generative-type cells in male and female lines with atypical features appearing during development in low temperature conditions.

## 2. Material and Methods

### 2.1. Plant Material

*B. perennis* L. seeds were collected from a natural habitat in Lublin (Poland). The seeds were sown in pots in September 2018 and placed in a greenhouse. In March 2019, the plants were divided into two groups and cultivated in controlled conditions for 3 weeks. One group was kept at a temperature of 18 °C, while the other plants grew at 4 °C. Optimal moisture was constantly maintained in the pots. After three weeks of cultivation, buds and opened inflorescences were collected at various stages of development. Individual flowers were isolated from the inflorescences and grouped according to the size. Freshly collected buds and flowers (50) at different stages of development were immediately fixed. The final selection of flowers was based on microscopic analysis.

### 2.2. Preparation of Material for Microscopic Studies

The material was prepared according to the procedures described previously by Wilson and Bacic [25]. The isolated flowers were placed in a fixative consisting of a 4% paraformaldehyde and 0.25% glutaraldehyde solution in 0.1 M PBS, pH 7.4, with the addition of a drop of Tween 80. For the first hour, the material was fixed under vacuum with the use of a vacuum pump. Afterwards, the material was transferred into a fridge for 24 h. After fixation, the material was rinsed in 0.1 M PBS, pH 7.4, twice for 3 h and then three times for 20 min. After rinsing, the material was dehydrated in increasing concentrations of ethanol: 30%, 40%, 50%, 60%, and 70% for 10 min at each concentration. The dehydration was continued in an increasing acetone series: 70%, 80%, and 90% to dry acetone (obtained by addition of 0.7 g of CuSO_4_ per each 10 mL of acetone). The material was dehydrated for 15 min in the 70%, 80%, and 90% acetone solutions and twice for 30 min in dry acetone. The dehydrated material was gradually saturated with the LR-White (Sigma Aldrich, Saint Louis, MO, USA) resin. The material was placed in acetone and polymer mixtures in the proportions of 3:1, 1:1, and 1:3 for 24 h in each mixture at 40 °C. Next, it was saturated with pure LR White twice for 24 h at 40 °C. The saturated flowers were placed in gelatin capsules, which were filled with the polymer and sealed. Vertically arranged blocks were polymerized for 24 h at 60 °C. The polymer-embedded material was cut into 1 μm-sections with the use of an ultramicrotome (Leica Reichert Ultracut S, Wien, Austria).

For histochemical analyses of developmental stages, the sections obtained were stained with a 1% aqueous solution of Toluidine blue.

### 2.3. Immunocytochemical Reaction Procedure

The immunofluorescence labeling was performed as in our previous work [13]. The one μm-thick sections were placed on poly-l-lysine coated slides (Sigma Aldrich) to prevent their detachment during the reaction and circled with a liquid blocker PAP Pen (Daido Sangyo, Tokyo, Japan). The well-dried preparations were rinsed with deionized water twice for 15 min. Next, 1% BSA in PBS was applied to the preparations to block non-specific protein binding sites. After 30 min, the slides were rinsed with 0.1 M PBS buffer, pH 7.4. The primary antibodies were diluted with 0.1% BSA in a ratio of 1:20. A different primary antibody was applied to each slide and left for 24 h at 4 °C. After removal of the antibodies, the slides were washed with PBS buffer twice for 20 min and then twice for 10 min each time. Afterwards, all procedures were carried out in a dark room. For visualization of the reaction, incubation with the secondary FITC-conjugated antibody (Sigma Aldrich), diluted 1:200 in the same buffer, was carried out for 12 h at 4 °C in the dark. Then, preparations were washed with PBS buffer for 15 and 20 min and five times with deionized water for 15 min each. A drop of a fluorescence-preserving substance—Dako Fluorescent Mounting Medium (Sigma Aldrich) was applied to the preparations and covered with a cover slide. The control reaction followed the procedure without the addition of primary antibodies outlined above.

### 2.4. Primary Antibodies

The following monoclonal antibodies were used: JIM13 (rat IgM antibody with a molecular mass of 80–100 kDa recognizing β-d-GlcA-(1,3)-α-d-GalA-(1,2)-α-l-Rha bonds in the AGP sugar chains) [26], JIM15 (rat IgG2c antibody with a molecular mass of 80–100 kDa; epitopes recognized by this antibody have not been identified so far; nevertheless, the antibody is known to recognize epitopes in the AGP sugar chains other than those recognized by JIM13 and JIM14) [27], and MAC207 (rat IgM antibody with a molecular mass of 70–100 kDa recognizing α-GlcA-(1,3)-α-GalA-(1,2)-α-Rha bonds in the AGP sugar chains) [28]. The antibodies used were obtained from Plant Probes, Paul Knox Cell Wall Lab (University of Leeds, Leeds, UK) and the Complex Carbohydrate Research Center (University of Georgia, Athens, GA, USA).

### 2.5. Imaging

The photographic documentation was made with the use of a confocal laser scanning microscope (Zeiss Axiovert 200M equipped with an LSM 5 Pascal laser scanning head, Göttingen, Germany). The microscopic studies were performed in a minimum of 50 sections for every stage and treatment. The presented photographs are representative. All parameters (i.e., laser intensity, gain) were kept constant throughout the experiment. The excitation wavelength for the FITC was 492 nm, and the emission was collected at 518 nm. The sections were analyzed against autofluorescence. The figures were edited using the CorelDraw X6 graphics program (https://www.coreldraw.com, accessed on November 2011).

## 3. Results

### 3.1. Localization of AGPs Epitopes in the Ovules of B. perennis during Megasporogenesis and Megagametogenesis

**Figure 1:**
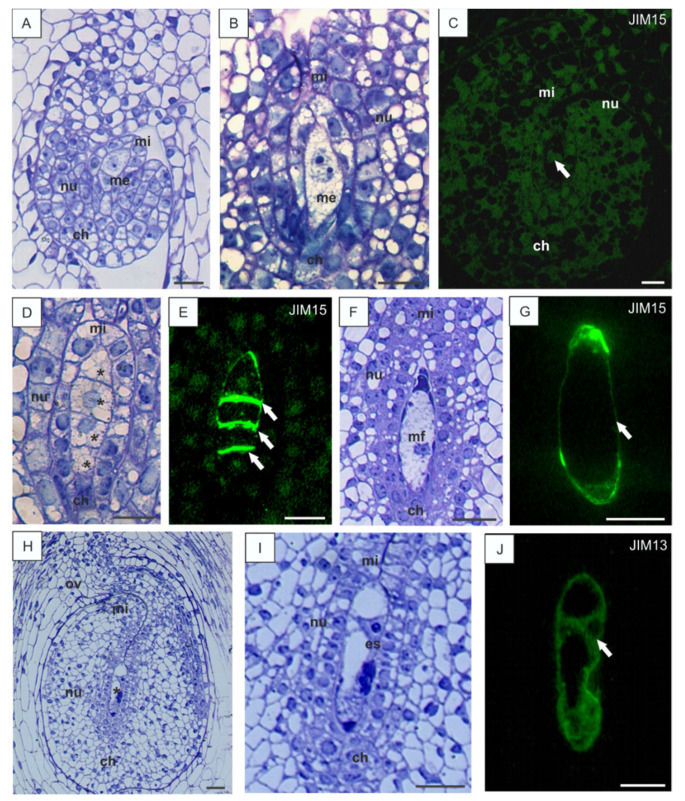
Histology of *B. perennis* ovules and immunolocalization of arabinogalactan proteins (AGPs) epitopes during megasporogenesis. Beginning of the differentiation process of the *B. perennis* ovule (**A**). Megasporocyte stage (**B**). Early stage of ovule development without visible presence of AGPs; reaction with JIM13; the arrow indicates megasporocyte (**C**). Tetrad of megaspores (asterisks) (**D**). AGP distribution in transverse walls between megaspores (arrows); reaction with JIM15 (**E**). Ovule with a functional megaspore (**F**). AGPs in the cell wall of the functional megaspore (arrow); reaction with JIM15 (**G**). Ovary with an ovule at the embryo sac stage (**H**). Magnification of a forming embryo sac (**I**). AGPs in the cell wall and cytoplasm of the embryo sac (arrow); reaction with JIM13 (**J**). Toluidine blue staining: (**A**,**B**,**D**,**F**,**H**,**I**). Immunolabeling: (**C**,**E**,**G**,**J**). Bars: 10 µm (**A**–**J**). Abbreviations: ch—chalazal pole, es—embryo sac, me—megasporocyte, mf—functional megaspore, mi—micropylar pole, nu—nucellus, ov—ovary wall.

At the early developmental stages of ovule development, the meristematic primordium grew and, at the beginning, formed a funiculus, which turned 90° to make the future ovule anatropous. Next, the integument was formed and, after some time, the megasporocyte surrounded by one layer of nucellar cells was differentiated. The fully developed ovules of *B. perennis* were anatropous, with one integument composed of several layers of cells (Figure 1A). The inner layer of integumental cells differentiated into the integumental tapetum. The integument grew, covered the megasporocyte and the layer of nucellar cells on the micropylar pole, and started to form the micropylar canal (Figure 1B). No AGP epitopes were observed in somatic or megasporocyte cells in the *B. perennis* ovules at the early developmental stages (Figure 1C). The megasporocyte grew and elongated in the micropylar-chalazal axis (Figure 1B). The large nucleus of the megasporocyte underwent the first meiotic division and the dyad was formed. After the second meiotic division, the linear tetrad of megaspores was formed. At the tetrad stage, the cells of integumental tapetum enlarged (Figure 1D). JIM15 epitopes appeared at the end of megasporogenesis in the transverse walls of the linear tetrad of megaspores (Figure 1E). The chalazal megaspore enlarged and developed into the embryo sac, while three other megaspores degenerated (Figure 1F). The AGP epitopes revealed with JIM15 were observed in the developing functional megaspore wall (Figure 1G). During the embryo sac development, the cells of the integumental tapetum lined the micropylar canal. Other cells of the integument were strongly vacuolized, with the exception of cells localized near the developing embryo sac, and contained denser cytoplasm (Figure 1H). During megagametogenesis, the haploid nucleus divided via mitotic division and a binucleate embryo sac was formed (Figure 1I). The presence of AGP epitopes was observed in the developing embryo sac. An intensive signal of fluorescence was visible in the embryo sac cell wall and in its cytoplasm (Figure 1J).

**Figure 2:**
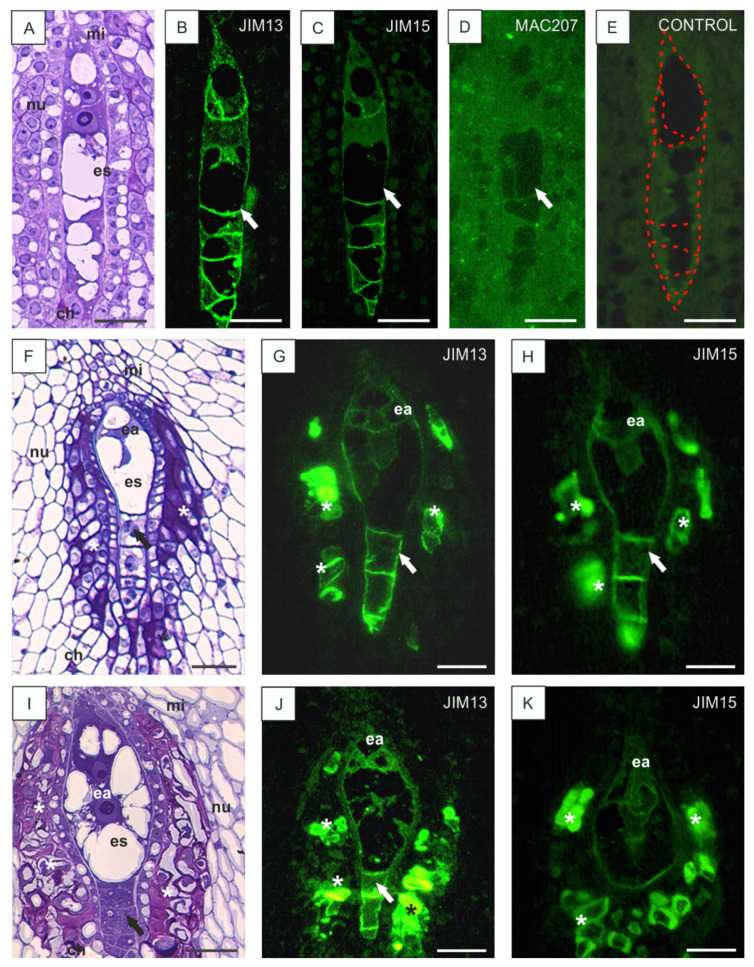
Histology of *B. perennis* ovules and immunolocalization of AGP epitopes during embryo sac development. Ovule nucellus with an embryo sac at an early stage of development (**A**). AGPs in the cell walls of the embryo sac (arrow); reaction with JIM13 (**B**), JIM15 (**C**), and MAC207 (**D**). Marked embryo sac (**E**). Later stage of embryo sac development than that shown in (**A**–**E**) (**F**). AGPs in the cell walls of the embryo sac and in cells surrounding the female gametophyte (asterisks); reaction with JIM13 (**G**) and JIM15 (**H**). Mature embryo sac at advanced stages of development (**I**). AGPs in the cell walls of the embryo sac and egg apparatus, reaction with JIM13 (**J**) and JIM15 (**K**). In (**F**–**J**) arrows point antipodal cells. In (**G**,**H**,**J**,**K**) asterisks point the integumental tapetum. Toluidine blue staining: (**A**,**F**,**I**). Immunolabeling: (**B**–**E**,**G**,**H**,**J**,**K**). Bars: 20 µm (**A**–**K**). Abbreviations: ch—chalazal pole, ea—egg apparatus, es—embryo sac, mi—micropylar pole, nu—nucellus.

During the subsequent stages, each nucleus underwent kariokinetic division and, after cytokinesis, the embryo sac developed. At the early stage of development, the embryo sac was elongated and spindle-shaped (Figure 2A). Both epitopes (recognized by JIM13 and JIM15) appeared in the newly formed cell walls of the synergids, the egg cell, the central cell, and the antipodal cells. It is worth mentioning that both epitopes were completely absent from the somatic tissues of the ovule at this stage of embryo sac development (Figure 2B,C). The labeling with MAC207 yielded scattered fluorescence in all somatic tissues of the *B. perennis* ovules at all developmental stages, but it was always absent from the embryo sac cells (Figure 2D). The control reaction without incubation with the primary antibody was characterized by the absence of the fluorescence signal (Figure 2E).

At the maturation stage, the shape of the embryo sac changed (Figure 2F,I), and the mature embryo sac contained two synergids and a larger egg cell at the micropylar pole. The vacuoles in the synergids were located at their chalazal pole, while the vacuole in the egg cell occupied their micropylar pole. No distinctive filliform apparatus was observed in the synergids. The center of the embryo sac was occupied by the central cell with one big vacuole and two polar nuclei, which fused in *B. perennis* comparatively early and created a large diploid secondary nucleus with a distinctive nucleolus. The antipodal cells were formed on the chalazal pole of the embryo sac (Figure 2I). At the stage leading to the formation of fully mature ovules, JIM13 and JIM15 epitopes appeared in the cell walls of the embryo sac cells and additionally in the integumental cells surrounding the embryo sac (Figure 2G,H). Apparently, the integumental cells surrounding the mature embryo sac underwent degeneration (Figure 2F,I).

During further development, numerous antipodals were formed and the shape of the embryo sac changed drastically compared to the earlier developmental stages. It became wider in the center, as numerous new vacuoles were formed in the central cell, and narrower at the chalazal pole, where the small antipodal cells were devoid of vacuoles. In ovules with a fully developed embryo sac, the integumental cells surrounding the female gametophyte underwent lysis and an amorphous substance appeared (Figure 2I). The examined AGP epitopes were present in the embryo sac wall and in integumental tapetum cells (Figure 2J). Additionally, an intensive signal indicating the presence of AGPs was visible in the egg apparatus (Figure 2K).

### 3.2. Disturbances in AGP Localization in Developing Ovules Caused by Low Temperature

**Figure 3:**
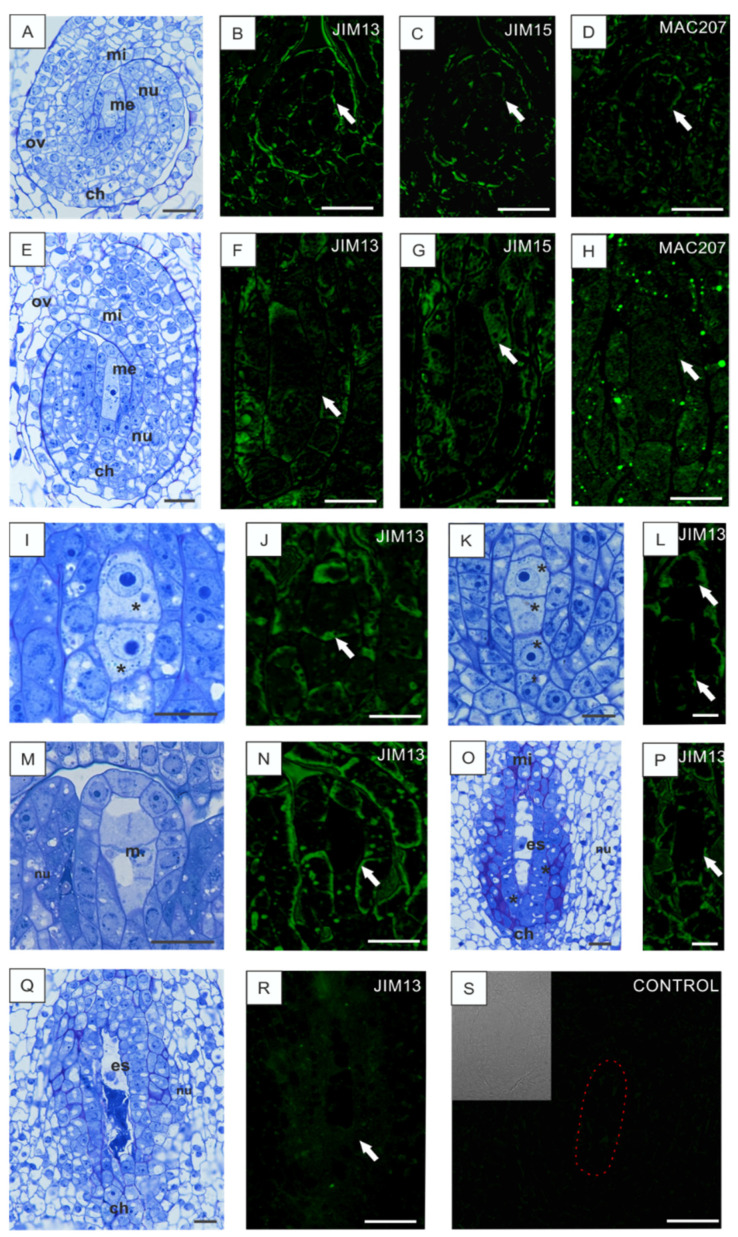
AGP localization in *B. perennis* ovules with disturbances in development caused by low temperature. Early stage of megasporocyte development (**A**). AGPs in the cell walls of the ovule (arrow); reaction with JIM13 (**B**), JIM15 (**C**), and MAC207 (**D**). Ovule with a formed megasporocyte (**E**). Epitopes of AGPs in the cell walls of adjacent megasporocyte cells of the nucellus and not visible in cell walls and cytoplasm of the megasporocyte (arrow); reaction with JIM13 (**F**), JIM15 (**G**), and MAC207 (**H**). Dyad stage (asterisks) (**I**). AGPs in dyad walls (arrow); reaction with JIM13 (**J**). Linear tetrad of megaspores (asterisks) (**K**). AGPs in the walls of megaspores (arrows); weak reaction with JIM13 (**L**). Stages of megaspore (**M**) and embryo sac formation (**O**). AGPs in the walls of somatic nucellus tissue (arrow); reaction with JIM13 (**N**,**P**). Degenerated embryo sac (**Q**). Absence of a fluorescence signal in the degenerated embryo sac (arrow); reaction with JIM13 (**R**). Control reaction with a marked embryo sac (**S**). Toluidine blue staining: (**A**,**E**,**I**,**K**,**M**,**O**,**Q**). Immunolabeling: (**B**–**D**,**F**–**H**,**J**,**L**,**N**,**P**,**R**,**S**). Bars: 20 µm (**A**–**H**), 10 µm (**I**–**S**). Abbreviations: ch—chalazal pole, es—embryo sac, m—megaspore, me—megasporocyte, mi—micropylar pole, nu—nucellus, ov—ovary wall.

In the subsequent part of our investigations, analyses of changes in the structures and immunolocalization of the specific AGP epitopes were performed in flowers growing under low temperature stress. The disturbances in the AGP occurrence at the advanced stages of development at low temperature and the degeneration of embryo sac are shown in Figure 3. At the early stage with a visible megasporocyte (Figure 3A,E), the AGP epitopes were distributed in the cell walls without noticeable specificity after incubation with JIM13 (Figure 3B), JIM15 (Figure 3C), and MAC207 (Figure 3D) and in the cell cytoplasm (Figure 3F–H). The fluorescence signal was dotted and accidental in both types of ovule cells, i.e., the megasporocyte and somatic cells. In the dyad of megaspores (Figure 3I,J) and the linear tetrad (Figure 3K,L), there was a very weak fluorescence signal in the transverse walls and a stronger signal in cells surrounding the megaspores. With the progress of the developmental process, which was based on an unusual dyad division and embryo sac formation (Figure 3M,O), the AGP epitopes were labeled with a similar pattern to that in the earlier stages, without typically increased fluorescence intensity in dividing cells (Figure 3N,P). An evident symptom of the disturbance caused by low temperature was the degenerated gametophyte (Figure 3Q), in which no fluorescence signal was observed with JIM13 and with the other two antibodies (Figure 3R). No fluorescence signal was noticeable in the sections with the control reaction (Figure 3S).

### 3.3. Low Temperature-Induced Disturbances in AGP Localization during Development of Anthers

**Figure 4:**
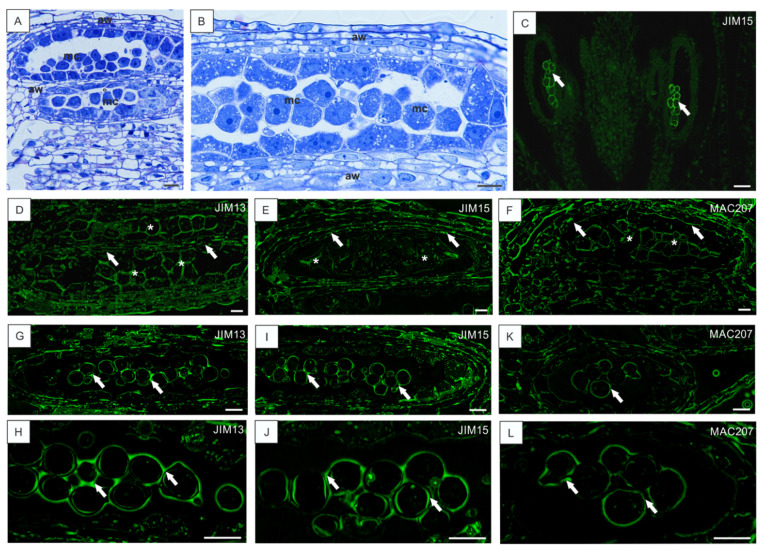
AGP localization in *B. perennis* anthers at early stages developing at low temperature. Histology of anther tissues during microsporocyte differentiation (**A**,**B**). Section of the whole anther with visible AGPs labeled in microsporocytes (arrows); reaction with JIM15 (**C**). AGPs in undifferentiated cell walls of the anther parietal layer (arrows) and microsporocytes in the anther loculus (asterisks); reaction with JIM13 (**D**), JIM15 (**E**), and MAC207 (**F**). AGPs in walls around microsporocytes (arrows) during the first meiotic division and in somatic cell walls; reaction with JIM13 (**G**,**H**), JIM15 (**I**,**J**), and MAC207 (**K**,**L**). Toluidine blue staining: (**A**,**B**). Immunolabeling: (**C**–**L**). Bars: 10 µm (**A**,**B**), 20 µm (**C**), 10 µm (**D**–**L**). Abbreviations: aw—anther walls, mc—microsporocyte.

**Figure 5:**
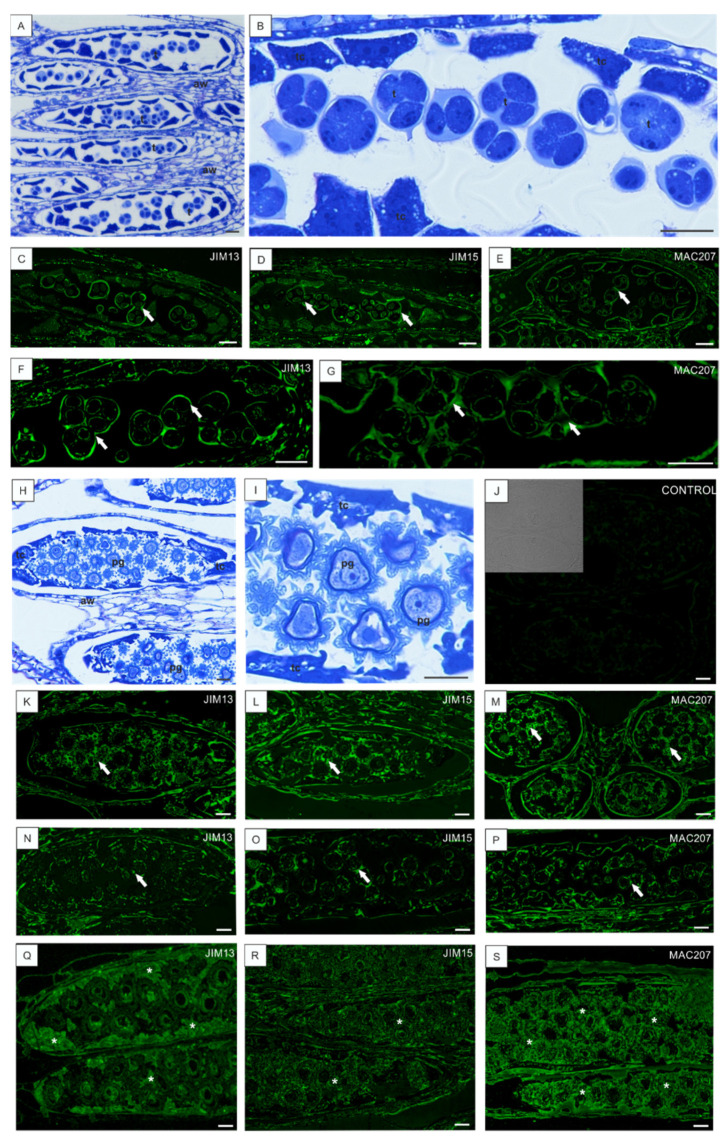
AGP localization in the *B. perennis* anther at the tetrad stage and with mature pollen grains developing at low temperature. Histology of anthers with tetrads of microspores (**A**,**B**). AGPs in walls around the microspores in the tetrad stage (arrows); reaction with JIM13 (**C**), JIM15 (**D**), and MAC207 (**E**). Higher magnification of tetrad microspores (arrows) labeled with JIM13 (**F**) and MAC207 (**G**). Histology of anthers filled by numerous mature pollen grains (**H**). Magnification of pollen grains with a developed exine layer (**I**). Control reaction to immunolabeling (**J**). Sections of anthers with pollen grains (arrows) and visible tapetum cells labeled by JIM13 (**K**), JIM15 (**L**), and MAC207 (**M**). Labeling of mature pollen grains (arrows) with JIM13 (**N**), JIM15 (**O**), and MAC207 (**P**). Immunofluorescence reactions of remnants of tapetum cells (asterisks) after reaction with JIM13 (**Q**), JIM15 (**R**), and MAC207 (**S**). Toluidine blue staining: (**A**,**B**,**H**,**I**). Immunolabeling: (**C**–**G**,**J**–**S**). (**M**)—transversal section; (**A**–**L**,**N**–**S**)—longitudinal sections. Bars: 10 µm (**A**–**S**). Abbreviations: aw—anther walls, pg—pollen grain, t—tetrads of microspores, tc—tapetum cells.

In our previous article [12], the distribution of AGPs during male gametophyte development in optimal conditions was analyzed. The temporal and spatial localization of AGPs at different developmental stages using JIM13, JIM15, and MAC207 were described. In the current work, we focused on the occurrence of AGPs during anther tissue differentiation in low temperature conditions (Figure 4 and Figure 5). In anthers with no significant signal of disruption during development, the distribution of AGP epitopes at the early stages of differentiation was not changed in comparison to the development in the optimal conditions (described in detail in the publication by Chudzik and coworkers [12]). The immunolabeling of sections with the whole anther showed that microsporocytes differed markedly in the intensity of the fluorescence signal from other cells (Figure 4C). At the early stages of differentiation of microsporocytes (Figure 4A,B), AGPs recognized by JIM13, JIM15, and MAC207 were noticeable in both the somatic cells of the anther wall and in the cells inside the loculus (Figure 4D–F). With the progress of microsporogenesis, the signal was more specific and strong in tapetal cells (Figure 4G,I,K). The higher magnification of the sections revealed very strong fluorescence in the common wall surrounding the microspores (Figure 4H,J,L). There were no differences in the fluorescence after application of the particular antibodies.

At the microspore tetrad stage before and after the cellularization process (Figure 5A,B), the fluorescence signal was visible in cell walls separating each tetrad of microspores (Figure 5F,G) and in the cytoplasm of tapetal cells (Figure 5C–E). In the anthers filled with released microspores and pollen grains at different stages of development, the surrounding walls underwent lysis (Figure 5H,I) and were labeled by the antibodies against AGPs (Figure 5K–M). The AGP epitopes were labeled in mature pollen grains with developed sporoderm layers, but the signal was weaker, and it was clearly visible that it began to disappear. Similarly, a dotted fluorescence signal was observed in the anther wall tissues (Figure 5N–P). Moreover, considerable amounts of adjacent remnants of tapetum cells were strongly labeled by each antibody used (Figure 5Q–S). The control reaction confirmed the proper labeling results (Figure 5J).

## 4. Discussion

AGPs are often reported to play important roles in sexual plant reproduction, including the male and the female gametophyte development, e.g., support of the pollen tube growth during the progamic phase and embryogenesis. Specific AGP epitopes are considered as molecular markers in sexual reproduction [11,29]. However, the distribution of AGP epitopes recognized by different available monoclonal antibodies differs significantly between tissue/cell types and plant species. The presence of specific epitopes in different cell types shows dynamic turnover during developmental processes. The aim of the current study was to describe the localization of AGPs in female and male structures developed upon cold temperature, and to examine the possible participation of AGPs in the response to stress.

In optimal conditions, AGP epitopes were detected in the generative organ at different developmental stages, but the JIM13 and JIM15 epitopes were very specific for the cells of male and female gametophytes. Another essential difference was the localization of the MAC207 epitope. In *B. perennis*, it was clearly accumulated in the cytoplasm of the vegetative cell of mature pollen grains. Accumulation of specific AGP epitopes recognized by different monoclonal antibodies was already detected in tissues connected with sexual reproduction in many plant species. The occurrence of AGP epitopes (JIM13, JIM15, LM2, MAC207) in the micropylar pole of the nucellus, in the embryo sac, and in the egg apparatus of *Amaranthus hypochondriacus* [30], *Arabidopsis thaliana* [11], *Olea europaea* [31], *Fragaria* × *ananassa* [13], and *Utricularia* [32] was described as the presumed role of AGPs during female structure development. AGPs were connected with active regulation of selection of megaspores [33], ‘promotion’ of viable megaspores [15], involvement in initiation of female gametogenesis [17], biochemical support for the pollen tube to reach the embryo sac in transmission track [32] and contribution to the fertilization, and the proembryo development [14,34]. Furthermore, AGPs occurred in mucilage mother cells, and their accumulation was correlated with a hypothetical role in communication between maternal tissues and the embryo [35]. The most often used antibody—JIM13—was detected in the tapetal anther layers of *Brassica napus* [36] and *Quercus suber* [37], in mature pollen grains, and in the wall of generative and sperm cells [38]. All these locations of AGPs were correlated with their assumed functions in microspore differentiation [36], involvement in microgametogenesis [37], regulation of microspore reprogramming and embryogenesis [38].

It is worth mentioning that we found that low temperature stress exerted an effect on the presence of AGPs at particular stages of *B. perennis* development. Briefly, the disturbances in the typical pattern of AGP distribution were observed both in female and in male lines. At the first stages of megasporogenesis, the AGP epitopes were arranged in an irregular, dotted, very accidental mode. No accumulation in specific cell structures was observed. Moreover, AGPs were present both in somatic and in generative cells. In turn, a significant increase in AGP epitopes occurrence only in generative cells was noticeable during development in typical conditions. The observations of the development of the female lines showed that low temperature conditions caused disorders, including degeneration of generative cells, i.e., the embryo sac. Given the role of AGPs as a marker of a specific stage of development, such as a ‘signal’ of initiation of megasporogenesis and a marker of newly formed female gametophyte that is ready for fertilization [11,13,16], this is even more important and allows assuming that the female gametophyte is not proper and not ready for the subsequent phases of development. In addition, the absence of AGP epitopes during the next stages of development could be crucial, as AGPs are proposed as nutrition material for the developing embryo sac and the embryo.

The distribution of AGPs during male gametophyte development in optimal conditions was described as very specific [12]. To sum up, JIM13 and JIM15 epitopes were detected to have a similar pattern in differentiated microsporocytes, walls surrounding dyads and tetrads of microspores, and tapetal cells. Strong fluorescence was visible in the continuous layer around microsporocytes, which allows a conclusion that this reflected accumulation of AGP epitopes. Interestingly, no AGPs recognized by the antibodies used were observed after the release of the microspores from the common walls; additionally, the fluorescence signal disappeared in tapetal cells and in mature pollen grains. In the case of MAC207 labeling, the pattern was the reverse of the JIM13 and JIM15 and was characterized by uniformly distributed epitopes localized even in different somatic tissues of the flower bud [12]. In the case of the development of male structures, the low temperature contributed to premature degeneration of tapetum cells in anthers. The tapetum degeneration was initiated at the stage of early differentiation. In the anthers from winter flowers, a complete absence of the tapetum was observed in the mature pollen stage, but the anther chambers were filled with abundant intercellular substance, which was not observed in the typical development. In anthers originating from the unfavorable climatic conditions with a degenerated tapetum, more intense and more abundant signal fluorescence indicating the presence of AGPs in tapetum remnants was visible.

The next step of our work was to find the cause of the changes in the AGP localization as an effect of cold temperature during the developmental process. We focused on the most significant modifications: disturbance in the AGP presence at the advanced stage—in the degenerated embryo sac and on the AGP accumulation in the tapetum remnants. Ma and Zhao [39] have shown that abiotic stress, such as low temperature, is correlated with differential regulation of *AGP* gene expression. Cold stress caused up-regulation of the *OsAGP3, OsAGP24,* and *OsAGP20* genes, which are connected with the role of their carbohydrate chains in the response to chilling. The polysaccharide chains of AGPs that can be deglycosylated might be a source of oligosaccharides and, in turn, might ‘increase the intracellular osmotic pressure and reduce the speed of dehydration’ [39]. Also, the involvement of AGPs in the tolerance of banana to chilling stress was investigated by Yan and coworkers [40]. The authors observed specific accumulation of AGP epitopes in cell walls and described it as a common phenomenon in banana leaves upon mild low-temperature treatment. The authors assume that this may be connected with the AGP function in the maintenance of cell turgor under low temperature stress and protection of banana against chilling injury through reinforcing mechanical strength and integrity of cell wall [40].

## 5. Conclusions

In our study on female and male *B. perennis* development, two distinct results were observed. In the case of the female structure, the changes are more radical and the influence of cold temperature on disturbance in the course of development is noticeable. The disorder manifests itself as anatomical changes, i.e., premature degeneration of the embryo sac and the absence of subsequent stages of megasporogenesis. Also, there are modifications in the male type line, such as accumulation of intercellular substances with tapetum remnants in the anther chamber. The distribution of AGPs is connected with these anatomical changes. The disorder is associated with lack of AGP in the cell wall, and it may be assumed that there are irregularities in the AGP synthesis.

## Data Availability

The data presented in this study are available on request from the corresponding author.
